# Effect of Deep Brain Stimulation on Regional Cerebral Blood Flow in Patients with Medically Refractory Tourette Syndrome

**DOI:** 10.3389/fpsyt.2016.00118

**Published:** 2016-07-05

**Authors:** Cathleen Haense, Kirsten R. Müller-Vahl, Florian Wilke, Christoph Schrader, Holger H. Capelle, Lilli Geworski, Frank M. Bengel, Joachim K. Krauss, Georg Berding

**Affiliations:** ^1^Department of Nuclear Medicine, Hannover Medical School, Hannover, Germany; ^2^Clinic of Psychiatry, Social Psychiatry and Psychotherapy, Hannover Medical School, Hannover, Germany; ^3^Department of Radiation Protection and Medical Physics, Hannover Medical School, Hannover, Germany; ^4^Department of Neurology, Hannover Medical School, Hannover, Germany; ^5^Department of Neurosurgery, Hannover Medical School, Hannover, Germany

**Keywords:** Tourette syndrome, deep brain stimulation, brain perfusion, ^99m^Tc-ECD-SPECT, prospective study

## Abstract

In this study, alterations in brain perfusion have been investigated in patients with Tourette syndrome (TS) compared with control subjects. In addition, we investigated the effects of deep brain stimulation (DBS) in both globus pallidus internus (GPi) and centromedian-parafascicular/ventralis oralis internus nuclei of the thalamus (CM/Voi) and sham (SHAM) stimulation on cerebral blood flow. In a prospective controlled, randomized, double-blind setting, five severely affected adult patients with TS with predominant motor or vocal tics (mean total tic score on the Yale Global Tic Severity Scale: 39) underwent serial brain perfusion single photon emission computed tomography with ^99m^Tc-ECD. Results were compared with data from six age-matched control subjects. All patients were investigated at four different time points: once before DBS implantation (preOP) and three times postoperatively. Postoperative scans were performed in a randomized order, each after 3 months of either GPi, CM/Voi, or SHAM stimulation. At each investigation, patients were injected at rest while awake, but scanned during anesthesia. This procedure ensured that neither anesthesia nor movement artifacts influenced our results. Control subjects were investigated only once at baseline (without DBS or anesthesia). At baseline, cerebral blood flow was significantly reduced in patients with TS (preOP) compared with controls in the central region, frontal, and parietal lobe, specifically in Brodmann areas 1, 4–9, 30, 31, and 40. Significantly increased perfusion was found in the cerebellum. When comparing SHAM stimulation to preOP condition, we found significantly decreased perfusion in basal ganglia and thalamus, but increased perfusion in different parts of the frontal cortex. Compared with SHAM condition both GPi and thalamic stimulation resulted in a significant decrease in cerebral blood flow in basal ganglia and cerebellum, while perfusion in the frontal cortex was significantly increased. Our results provide substantial evidence that, in TS, brain perfusion is altered in the frontal cortex and the cerebellum and that these changes can be reversed by both GPi and CM/Voi DBS.

## Introduction

Tourette syndrome (TS) is a neuropsychiatric disorder characterized by the presence of chronic, fluctuating motor and vocal tics. It is associated with an increased risk of comorbid emotional and behavioral psychopathologies, including attention deficit hyperactivity disorder (ADHD) and obsessive–compulsive disorder (OCD), which considerably affect one’s individual prognosis (1). Although it is believed that TS is an inherited condition, the precise underlying genetics still remain unknown (2).

The neurobiology and the pathomechanisms of TS are still not completely understood (3). Some studies show a predominant involvement of cortico-striato-thalamo-cortical (CSTC) circuits with links from distinct frontal cortical regions to subcortical structures (4, 5). Tics are thought to be secondary to focal excitatory abnormalities in the striatum, which lead to an erroneous inhibition of a group of neurons in the globus pallidus internus (GPi) and, on the other hand, to a disinhibition of cortical neurons (6, 7). Accordingly, neuropathological studies have reported on cellular alterations in the basal ganglia, such as an increased number of neurons in the GPi along with a reduction of quantity and density of neurons in the globus pallidus externus and nucleus caudatus (8). In addition, decreases in volume and microstructural changes in the thalamus have been found (9).

Alternatively, a primary cortical dysfunction has been suggested, supported by different structural and functional neuroimaging studies. For example, hypoperfusion in frontal cortex areas, including prefrontal and premotor frontal cortices as well as the primary motor cortex, has been reported in patients with TS with mild to moderate tics using ^15^O-H_2_O positron emission tomography (PET) (10). In line with these data, volume reductions in frontal regions, reduced prefrontal cortical thickness, and abnormal gray matter diffusivity in the orbitofrontal cortex have been reported (11, 12). Recent magnetic resonance imaging (MRI) studies, in addition, provided evidence for an involvement of the SMA in tic generation (13–15). However, until today, it is unclear which of these abnormalities are related to the underlying cause of the disease and which are due to secondary compensatory effects.

Treatment of patients with TS is difficult and often unsatisfactory. Pharmacological interventions, including antipsychotics, clonidine, botulinum-toxin injections as well as cannabinoids, neither cover the complete spectrum of symptoms nor target additional behavioral problems, adequately (16). Furthermore, available therapies are often associated with intolerable side effects. Therefore, deep brain stimulation (DBS) has been suggested as an alternative treatment for medically refractory, severely affected, adult patients with TS (17). Stimulation of various targets, including the centromedian-parafascicular/ventralis oralis internus nuclei of the thalamus (CM/Voi) as well as the GPi, resulted in beneficial clinical effects with tic improvement and variable amelioration of comorbidities, such as OCD, anxiety, and self-injurious behavior (18–22). However, the underlying mechanisms of DBS in TS and its influence on abnormal cerebral perfusion remain unknown.

To the best of our knowledge, this is the first study investigating the impact of bilateral DBS of both the GPi and the CM/Voi on regional cerebral blood flow in severely affected, medically refractory, adult patients with TS. In order to image these severely affected patients, serial single photon emission computed tomography (SPECT) with ^99m^Tc-ECD was employed. This technique is feasible since the radiotracer is injected in the awake state and distributes according to the cerebral perfusion at the time of injection. Subjects may then be anesthetized for motion-free imaging of cerebral perfusion at the time of radiotracer injection, but not at the time of imaging.

## Materials and Methods

### Subjects

In this prospective controlled, randomized, double-blind study, five severely affected adult patients with TS according to DSM-IV-TR criteria were enrolled (three women, two men, mean age ± SD, 29 ± 11, range, 19–47 years). Patients had to present with predominant and severe motor or vocal tics [total tic score (TTS) of the Yale Global Tic severity Scale (YGTSS) >35] (23). Prior interventions with at least three different medications (e.g., typical and atypical antipsychotics) must have had failed to improve the tics or resulted in intolerable side effects. Eight weeks before study entry and during its complete course, medication for the treatment of TS remained stable. Medication included antipsychotics (*n* = 4), serotonin reuptake inhibitors (*n* = 2), benzodiazepines (*n* = 1), and anticholinergics (*n* = 1). One patient was free of any neurotropic medication.

Brain perfusion studies of patients were compared with those of six control subjects (one woman, five men, mean age ± SD, 43 ± 8, range, 30–52 years), which represented neuropsychiatrically healthy subjects with tumors of the skull base, neck, or throat. TS patients and controls did not significantly differ with respect to age and gender. All control subjects gave written-informed consent receiving baseline brain perfusion scans before surgery. All patients gave written-informed consent to participate in the clinical study and, additionally, in the present imaging study. Both studies were approved by the local ethics committee of Hannover Medical School, and the imaging study also by the German Federal Office for Radiation Protection (trial registration identifier: Z5-22461/2-2008-006). Patients received extensive screening, clinical examinations, and a diagnostic battery consisting of neurologic, psychiatric, neuropsychological, and structural MRI examinations to exclude diseases other than TS.

### Clinical Assessment

All patients underwent a detailed clinical and neuropsychological evaluation. The YGTSS was used as a semi-structured clinical rating instrument to evaluate the number, frequency, intensity, complexity, and interference of motor and vocal tics and to indicate disease severity (23). Comorbid emotional and behavioral psychopathologies in our patients included alcoholism (*n* = 1), conduct disorder (*n* = 1), OCD (*n* = 1), subclinical OCD (*n* = 1), anxiety disorder (*n* = 1), and major depression (*n* = 1). None of our patients suffered from ADHD at time of investigation. Upon final conclusion of the clinical study with 10 patients, clinical data will be given in detail elsewhere.

### Deep Brain Stimulation

**Figure 1:**
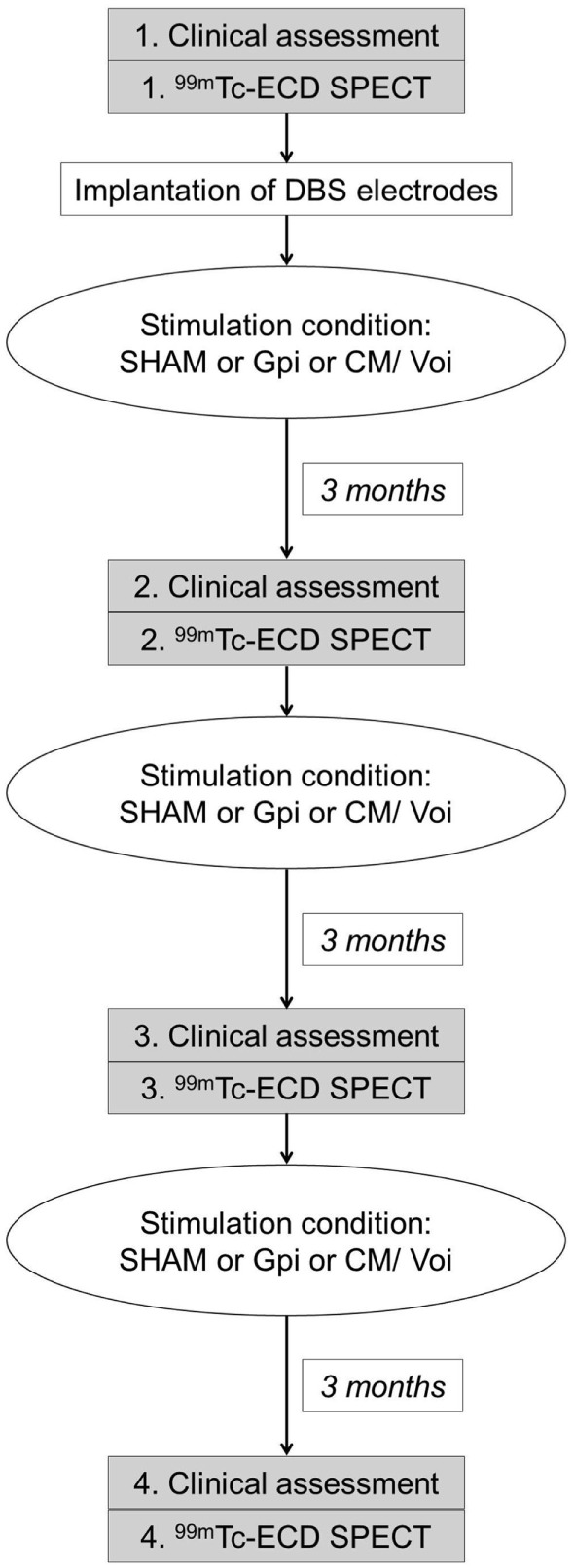
**Flow chart illustrating the study design**.

Patients with TS underwent presurgical preparation in the Department of Neurosurgery. DBS electrodes (Medtronic 3387) were placed both in the posteroventral lateral GPi and in the thalamic CM/Voi bilaterally, during general anesthesia in the frame of the clinical study protocol. Electrode placement was guided *via* CT-stereotactic surgery refined by microelectrode recording. In a second step, a dual channel implantable pulse generator (Kinetra, Medtronic) was implanted in the subclavicular region with a switch allowing to connect all four electrodes. Stimulation conditions of the electrodes (SHAM, GPi, and CM/Voi) were applied in a randomized order according to the study protocol (Figure 1). Prior to programing of DBS settings, thresholds for any stimulation-induced side effects were determined to allow blinding of the patient with subsequent subthreshold chronic stimulation. Each condition lasted 3 months to allow for stable adjustment. Patients and clinical investigators were blinded to the stimulation condition.

### ^99m^Tc-ECD SPECT

Brain perfusion studies were performed in accordance with the guidelines of the European Association of Nuclear Medicine (EANM) (24). Before injection of ^99m^Tc-ethyl-cysteinate-dimer (^99m^Tc-ECD, Neurolite^®^, IBA/CIS bio GmbH, Berlin, Germany), patients were lying comfortable for 15 min in a quiet room with dimmed light. They were instructed not to speak and to relax but not to suppress their tics during this time and for an additional 5 min after application of the radiopharmaceutical. Controls were injected under the same conditions.

Single photon emission computed tomography scanning was performed 1 h after injection of 550 MBq ^99m^Tc-ECD using a dual head camera (ECAM variable, Siemens, Erlangen, Germany). The participants were positioned supine and with the canthomeatal line perpendicular to the rotation axis. Patients were scanned during anesthesia to allow for motion-free acquisition. Controls were studied without anesthesia, but as neuropsychiatrically healthy subjects they were readily able to avoid head movements. Patients were studied four times: once before DBS implantation (preOP) and three time after surgery, each after 3 months of either GPi DBS, CM/Voi DBS, or sham stimulation (SHAM), which were applied in a randomized order (Figure 1). Since ^99m^Tc-ECD was no longer commercially available in Germany, the enrollment into the imaging study had to be terminated after inclusion of five patients.

### Data and Statistical Analysis

3D datasets were spatially normalized into stereotactic standard space according to Montreal Neurological Institute (MNI) using the default brain perfusion template of SPM2 (Statistical Parametric Mapping, Wellcome Trust Centre for Neuroimaging, London, UK) and employing affine and non-linear procedures (16 non-linear iterations, 7 × 9 × 7 basis functions). 3D datasets were smoothed (FWHM 10 mm) and rescaled to the 75th intensity percentile of the whole brain. Rescaled 3D datasets of patients and control subjects were compared based on volumes of interest (VOIs) as well as voxelwise to detect regional changes of brain perfusion. VOIs were delineated by automated anatomical labeling using the Cyceron and the Brodmann (BA) map of the brain, respectively (25).

To identify changes in larger brain regions, small VOIs according to Cyceron were summarized to large VOIs as follows: (i) frontal lobe = superior, middle, inferior and orbital frontal gyrus, and supplementary motor area; (ii) parietal lobe = superior and inferior parietal gyrus, angular gyrus, and precuneus; (iii) temporal lobe = superior, middle and inferior temporal gyrus, Heschl’s gyrus, and temporal pole; (iv) limbic lobe = hippocampus and parahippocampus and amygdala; (v) cingulum = anterior, middle, and posterior cingulate gyrus; (vi) occipital lobe = cuneus, lingual gyrus and superior, middle, and inferior occipital gyrus; (vii) central region = precentral and postcentral gyrus and paracentral lobe; and (viii) cerebellum. Moreover, the following regions were evaluated separately: caudate, putamen, pallidum, and thalamus. Always the average of left and right side was considered. Mean rescaled counts in VOIs were compared between groups (preOP vs. controls) and conditions (e.g., GPi vs. SHAM) using *t*-tests for independent and paired samples with a threshold of at least *p* < 0.05 for significance, respectively. Voxelwise comparisons were done using SPM2 with combined thresholds for statistical inferences of *p* = 0.001 on voxel level and *p* = 0.01 on cluster level (uncorrected *p*-values).

## Results

### Clinical Assessment

There were no surgical complications. Postoperative stereotactic computed tomography (CT) confirmed DBS electrode placement in all patients. Before DBS (preOP), patients had a mean YGTSS-TTS (±SD) of 39 (±5). During all postoperative conditions, the mean YGTSS-TTS was reduced – GPi: 33 (±10), CM/Voi: 33 (±14), SHAM 31 (±13). There were no significant differences between conditions. Detailed results will be reported elsewhere after completion of the study including *n* = 10 patients.

### Cerebral Perfusion – Patients vs. Controls

In patients with TS preOP compared with control subjects, analysis of large VOIs showed significantly decreased cerebral perfusion in the central region, the frontal lobe, and the parietal lobe (Table 1). Cerebellar perfusion was significantly increased.

**Table 1:** **Significant differences in blood flow between patients preoperatively and control subjects**.

Direction of flow change	VOI analysis				SPM analysis			
	Large region	*p*	Brodmann area	*p*	Subregion	MNI (*x y z*)	Voxel	*Z* value
Decrease	*Central* cortex	0.0020	BA 1	0.0103	Right pre- and post*central*	36 −12 68	367	4.41
			BA 4	0.0275	Left para*central*	−14 −54 78	143	4.28
Decrease	*Frontal* cortex	0.0138	BA 6	0.0001	Bilateral *SMA*, superior *frontal*	−10 20 60	335	4.43
			BA 8	<0.0001	Right middle *frontal*	38 8 64	245	4.15
			BA 9	0.0012				
Decrease	*Parietal* cortex	0.0026	BA 5	0.0388	Bilateral precuneus	−6 −50 56	277	3.98
			BA 7	0.0090	Left superior *parietal*, inferior parietal, angular gyrus	−40 −64 60	253	4.48
			BA 40	0.0017	Right superior *parietal*	32 −54 68	146	4.10
					Right inferior *parietal*, angular gyrus	54 −62 46	47	4.29
Decrease			BA 30	0.0416				
			BA 31	0.0050				
Increase	*Cerebellum*	0.0079			Right *cerebellum*	16 −70 32	124	4.01
					Left *cerebellum*	0 −18 −34	153	3.57

With respect to BA, significant flow reductions were detected likewise in the central region (BA 1and BA 4), the frontal cortex (BA 6, including the SMA, BA 8, and BA 9), the parietal cortex (BA 5, BA 7, and BA 40), and in the posterior cingulum (BA 30 and BA 31).

**Figure 2:**
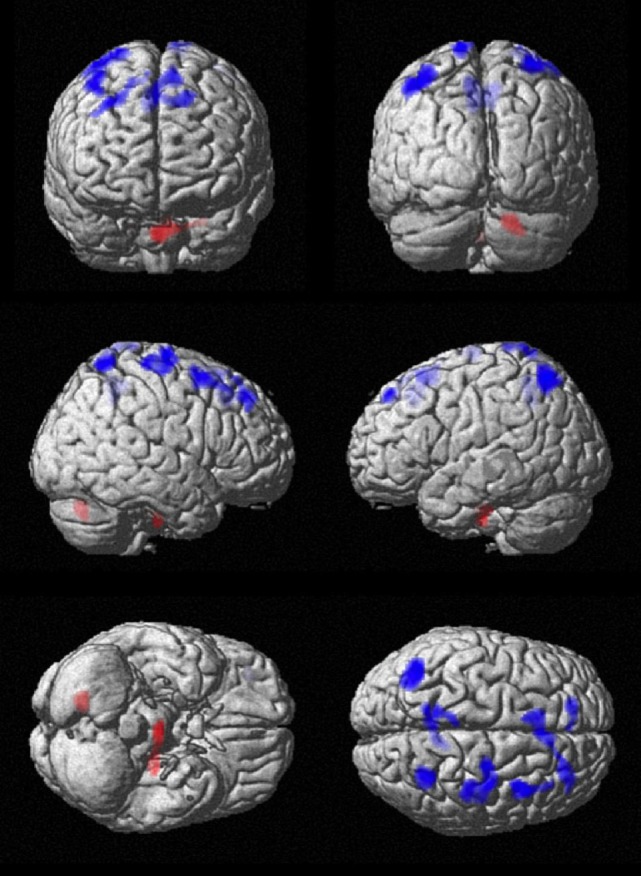
**Comparison of cerebral perfusion between patients and controls**. Statistical parametric map (extent threshold *k* = 124 voxel) projected onto surface display of MRT in MNI stereotactic space. Decreased blood flow (blue) in patients was evident in frontal, central, and parietal cortex, while increased flow (red) was present in the cerebellum.

SPM analysis showed correspondingly decreased perfusion in the central cortex (right pre- and postcentral, left paracentral), the frontal cortex (bilateral SMA, bilateral superior frontal cortex, and right middle frontal gyrus), and the parietal cortex (bilateral precuneus as well as bilateral superior, inferior and angular gyrus). Perfusion was increased in the left temporal cortex and bilaterally in the cerebellum. Results of SPM analysis are given in Table 1 and displayed in Figure 2.

### Cerebral Perfusion – SHAM vs. preOP

**Figure 3:**
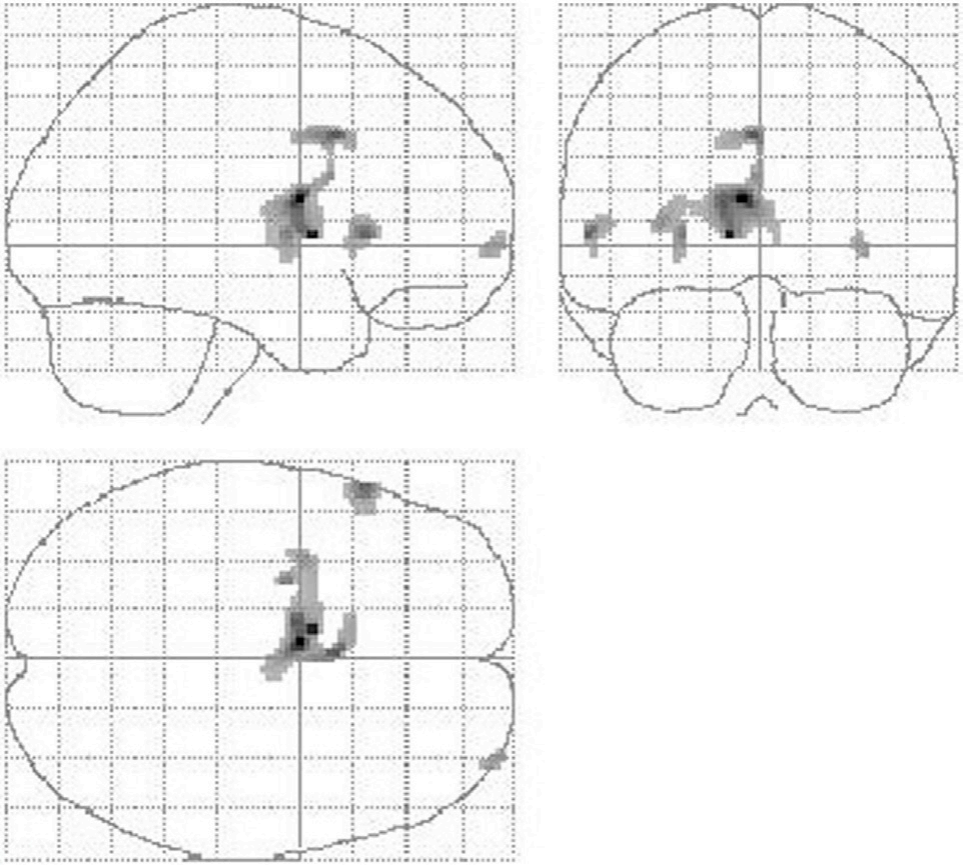
**Reduction of cerebral perfusion after DBS electrode placement during SHAM condition compared with preOP**. An extended flow reduction was observed encompassing multiple areas within basal ganglia and thalamus (extent threshold *k* = 23 voxel).

When comparing SHAM to preOP condition, VOI analysis showed decreased perfusion in the thalamus, putamen, pallidum, anterior and posterior cingulate (BA29, BA33), as well as inferior frontal cortex (BA44, BA45) (Table 2). SPM analysis, likewise, revealed reduced perfusion in the basal ganglia, thalamus, and cingulum (left caudate, putamen, anterior and middle cingulum, right thalamus), as well as frontal cortex (left inferior, right middle, and superior gyrus). Areas of decreased perfusion detected in SPM analysis are shown in Figure 3 and listed in Table 2. Increased perfusion during SHAM condition was found in VOI analysis only in the occipital cortex (BA17). Using SPM, we detected higher perfusion in the right SMA, temporal (superior, middle gyrus), and left occipital cortex.

**Table 2:** **Significant differences in blood flow between patients during SHAM condition vs. preoperatively**.

Direction of flow change	VOI analysis		SPM analysis			
	Region	*p*	Region	MNI (*x y z*)	Voxel	*Z* value
Decrease	*Thalamus*	0.0143	Left caudate, left *putamen*, right *thalamus*, left *anterior cingulate*	−6 0 16	420	4.78
	*Putamen*	0.0247				
	Pallidum	0.0308				
	BA 29	0.0383	Left middle cingulate	−2 12 38	66	4.28
	*BA 33*	0.0382				
Decrease	*BA 44*	0.0190	Left *inferior frontal cortex*	−58 22 4	55	4.20
	*BA 45*	0.0349				
Decrease			Middle and superior frontal cortex	34 66 0	13	3.66
Increase	BA 17	0.0372	Calcarine, cuneus	−4 −76 20	25	3.72
Increase			Right SMA, middle cingulate	14 −8 50	29	4.30
			Superior, middle temporal cortex	74 −30 0	42	4.06

### Cerebral Perfusion – GPi Stimulation vs. preOP and SHAM, Respectively

**Figure 4:**
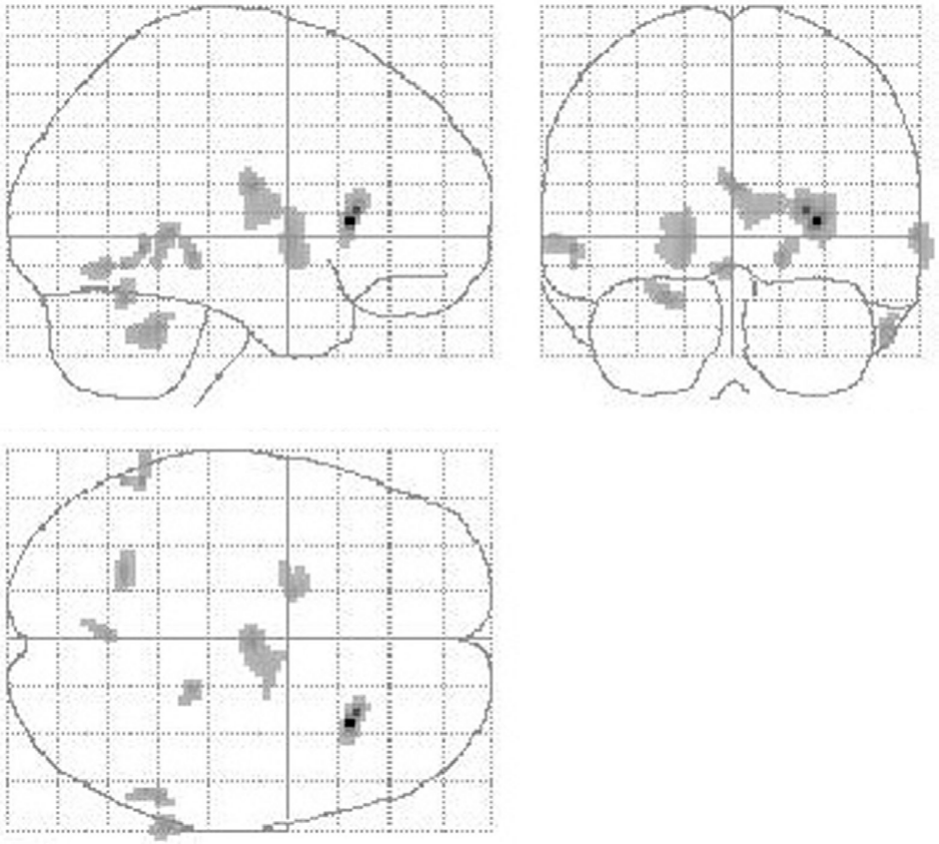
**Reduction of cerebral perfusion after DBS electrode placement during GPi stimulation compared with preOP**. Additionally to flow reductions in the basal ganglia and thalamus as detected during SHAM condition (see Figure 3) GPi stimulation resulted into reduced flow within the cerebellum (extent threshold *k* = 13).

During GPi stimulation, compared with preOP condition, blood flow was reduced according to VOI analysis in the thalamus, putamen, pallidum, and cerebellum (Table 3). SPM analysis largely confirmed lower perfusion in thalamus, basal ganglia, and cerebellum (right thalamus, caudate, putamen, insula, frontal inferior gyrus, left pallidum, putamen, cerebellum bilaterally) (Table 3). Moreover, perfusion was decreased in the temporal cortex (right middle gyrus, hippocampus, left middle gyrus). Figure 4 shows areas of reduced perfusion. No increases of perfusion were found.

**Table 3:** **Significant differences in blood flow between patients during GPi condition vs. preoperatively and SHAM condition**.

Comparison	Direction of flow change	VOI analysis		SPM analysis			
		Region	*p*	Region	MIN (*x y z*)	Voxel	*Z* value
GPi vs. preOP	Decrease	*Thalamus*	0.0365	Right *thalamus*	2 −12 18	147	4.12
	Decrease	*Putamen*	0.0187	Right caudate, *putamen*, insula, inferior frontal cortex	30 22 6	100	5.27
	Decrease	*Pallidum*	0.0167	Left *pallidum*, putamen	−16 2 0	126	3.90
	Decrease	*Cerebellum*	0.0418	Right *cerebellum*	56 46 −32	47	3.96
				Left *cerebellum*	−24 −58 −20	39	4.10
	Decrease			Right middle temporal cortex	66 −42 2	40	4.08
				Right hippocampus	18 −34 −6	27	4.04
				Left middle temporal cortex	−56 −52 −4	29	4.16
GPi vs. SHAM	Decrease	*Putamen*	0.0384	Right *putamen*	30 20 −2	46	3.82
				Left *putamen*, supra orbital frontal cortex, insula	−26 8 −10	40	3.30
	Decrease			Right caudate	10 10 −6	29	3.76
				Right thalamus	6 −14 8	20	3.64
	Decrease			Right cerebellum	48 −68 −24	48	3.86
				Left cerebellum	−24 −86 −32	24	3.93
	Decrease			Left inferior and middle temporal cortex	−46 −22 −38	75	4.79
				Left fusiform gyrus	−34 −60 −2	51	4.72
				Right temporal pole and inferior cortex	42 6 −36	31	3.65
				Left cuneus	−6 −82 18	32	3.93
	Increase			Superior and middle frontal cortex	28 42 44	39	3.65
				Precuneus bilateral	4 −52 44	20	4.17
				Bilateral middle and right anterior cingulate	0 −4 32	33	4.47

When comparing GPi stimulation to SHAM condition, again flow reductions in the basal ganglia, thalamus, and cerebellum were observed (VOI analysis: putamen, SPM analysis: right putamen, caudate, thalamus, cerebellum, left putamen, frontal supra orbital gyrus, insula, and cerebellum). Furthermore, perfusion was decreased in the temporal lobe (left inferior, superior, fusiform gyrus, right pole, inferior gyrus) and left cuneus (Table 3). Blood flow increases were seen in the right frontal cortex (superior, middle gyrus), bilateral precuneus, and bilateral middle and right anterior cingulum.

### Cerebral Perfusion – CM/Voi Stimulation vs. preOP and SHAM, Respectively

**Figure 5:**
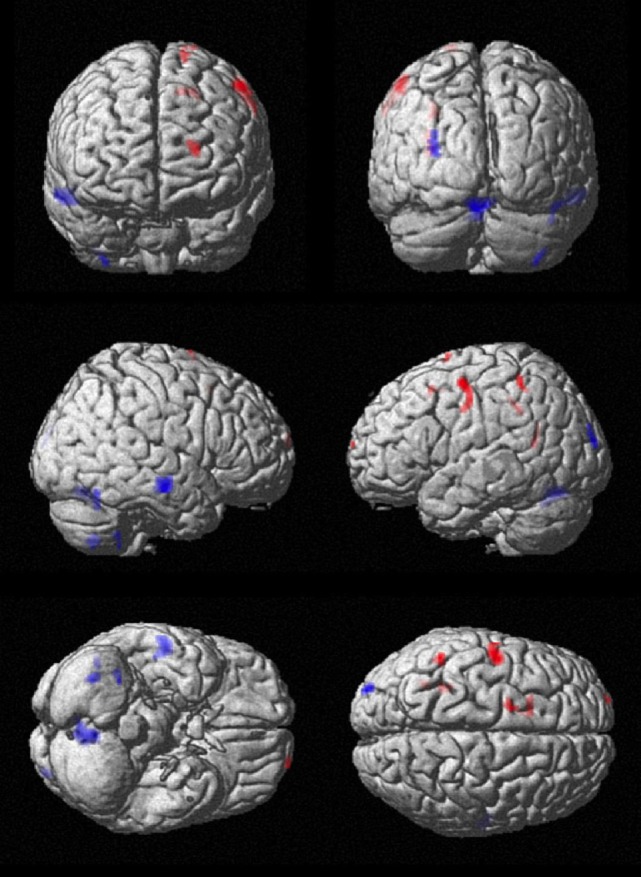
**Comparison of cerebral perfusion with CM/Voi stimulation vs. SHAM**. Effective stimulation resulted in flow reductions in the cerebellum and increases in the central and frontal cortex, specifically encompassing the SMA.

Comparing CM/Voi stimulation to preOP condition, SPM analysis showed reduced blood flow in the right caudate, left inferior frontal cortex, and right middle and superior temporal gyrus. Flow was increased in the left paracentral lobe, right pre-, post-, and supramarginal gyrus, and middle frontal gyrus (Table 4). Comparison of CM/Voi to SHAM stimulation showed flow decreases bilaterally in the cerebellum, left middle occipital gyrus, right middle and superior temporal gyrus, and right fusiform gyrus. Increased flow was detected in the left frontal superior gyrus, SMA and frontal middle gyrus, left pre- and postcentral gyrus, and the left inferior parietal and postcentral gyrus. Figure 5 shows blood flow changes during CM/Voi compared with SHAM stimulation detected with SPM. VOI analysis confirmed reduced flow in the cerebellum and occipital cortex (BA19), as well as increased flow in the frontal cortex (BA10) (Table 4).

**Table 4:** **Significant differences in blood flow between patients during CM/Voi condition vs. preoperatively and SHAM condition**.

Comparison	Direction of flow change	VOI analysis		SPM analysis			
		Region	*p*	Region	MIN (*x y z*)	Voxel	*Z* value
CM/Voi vs. preOP	Decrease			Right caudate	16 4 26	71	3.32
				Left inferior frontal cortex	−54 18 4	37	4.21
				Right middle and superior temporal cortex	50 −16 −14	24	3.94
	Increase			Left paracentral lobe	−10 −22 86	148	4.59
				Right pre-, post-, and supramarginal gyrus	46 −30 40	98	4.43
				Middle frontal cortex	−52 12 46	37	4.25
CM/Voi vs. SHAM	Decrease	*Cerebellum*	0.0055	Left *cerebellum*	−4 −68 −28	170	4.67
				Right *cerebellum*	46 −60 −20	23	3.70
	Decrease	*BA 19*	0.0486	Left middle *occipital cortex*	−28 −96 14	27	3.87
	Decrease			Right middle and superior temporal cortex	52 −16 −18	123	4.85
				Right fusiform gyrus	24 −62 −14	20	3.63
	Increase	*BA 10*	0.0012	Left superior *frontal cortex*	−18 0 78	45	4.05
				Left SMA and middle *frontal cortex*	−22 14 50	29	3.67
	Increase			Left pre- and postcentral cortex	−48 −6 56	82	4.29
				Left inferior parietal and postcentral cortex	−30 −44 38	26	4.26

## Discussion

We investigated regional cerebral perfusion patterns in patients with TS not only compared with neuropsychiatrically healthy control subjects but also during different conditions of DBS. To the best of our knowledge, this is the first neuroimaging study investigating TS patients suffering from extreme tics – those patients who are usually excluded from imaging studies due to unavoidable motion artifacts. Since image acquisition had been carried out during anesthesia, movement artifacts could be completely excluded. Anesthesia, however, did also not influence our image data, since tracer injection was done in awake condition. Finally, also influences by age and gender could be excluded, confirming that changes in cerebral perfusion patterns were due to the pathophysiology of patients with severe TS and not related to demographic characteristics.

Before implantation of the electrodes, patients with TS showed hypoperfusion in central regions, including the primary motor cortex and the postcentral gyrus, regions of the frontal cortex, including the superior and medial frontal gyrus, and the supplementary motor area, as well as parts of the parietal lobe when compared with control subjects. These findings are in line with previous reports of less affected patients demonstrating hypoperfusion in regions of the frontal cortex and the primary motor cortex using ^99m^Tc-ECD SPECT and ^15^O-H_2_O PET, respectively (10, 26). Using anatomical MRI, reduced cortical thickness of sensorimotor cortices as well as gray matter volume reduction of frontal regions could be demonstrated, pointing to a possible involvement of the frontal cortex in the pathology of TS (11, 27). In TS, it is assumed that especially regions of the frontal lobe and the central cortex are predominantly affected, which are involved in planning, controlling, and regulating the movements. Thus, from available data, it is suggested that, in TS, inhibitory mechanisms necessary for motor control are insufficient.

We also found an increased perfusion in the cerebellum in patients with TS compared with control subjects. This finding is in line with recent studies using ^15^O-H_2_O PET, ^18^F-fluorodeoxyglucose-PET, and event-related functional (f)MRI reporting about an involvement of the cerebellum in TS pathology (28–30). Since the CSTC network is supposed to play a major role in the pathophysiology of TS, the cerebellum has been suggested being a “second-in-line” structure affected by disturbed connections of the network (28). It has been hypothesized that the cerebellum is involved not only in motor execution and initiation of tics but also in the sensation of premonitory urges before the tics (28). Since the cerebellum has multiple connections to the basal ganglia and the thalamus, it has also been suggested that an overactivity of the cerebellum contributes not only to the origin of tics but also to common psychiatric comorbidities, such as ADHD and OCD (28). This hypothesis is supported by data obtained from patients suffering from pure OCD, which demonstrated increased gray matter volumes bilaterally in the anterior cerebellum (31). Furthermore, data from patients with pure ADHD showed reduced cerebellar activity using fMRI (32). Our findings of an increased cerebellar perfusion in TS preOP compared with control subjects, as well as a significant decrease of the cerebellar perfusion during both GPi and CM/Voi stimulation compared with SHAM condition further corroborate the hypothesis that the cerebellum is pathophysiologically involved in the primary cause of TS. Accordingly, the cerebellum has been suggested as a vitally important target for therapeutic interventions in TS (33).

To investigate the influence of the implantation of the electrodes *per se* on cerebral perfusion, we compared the SHAM condition with the preOP status. As expected, the main finding was a decrease of the cerebral blood flow in the target regions (GPi and CM/Voi). These findings are most likely correlates of a “microlesional effect.” Our results correspond to those reported by Hilker et al. (34) in patients with Parkinson’s disease, which demonstrated hypometabolism in the subthalamic nucleus during the “off condition” 6 months after implantation of electrodes in this target area (34). However, during SHAM condition, we also observed a unilateral increase of cerebral perfusion in the SMA (a brain area known to be relevant for motor control) suggesting that even the mere implantation of DBS electrodes (without stimulation) might influence TS pathophysiology. One can speculate that clinical improvement during SHAM stimulation – as detected in this study – might be related to such changes. Remarkably, tic severity was reduced both during the SHAM condition and during GPi and CM/Voi stimulation. We believe that it would be premature to draw any conclusions from these preliminary findings, which might be related to various issues, such as the low number of patients included, a placebo effect, the spontaneous fluctuations in tic severity, and the implications of subjectivity on tic assessments. We are confident to clarify this issue upon conclusion of the clinical study after inclusion of a larger sample size and assessing all available clinical evaluations, including video protocols and other measures.

During both GPi and CM/Voi stimulation, a more extended increase of cerebral perfusion (compared with SHAM condition) was found in different regions of the frontal cortex: in the right superior and middle frontal area, during GPi stimulation, and in the left superior as well as middle frontal gyrus and supplementary motor area, during CM/Voi stimulation. This finding could be interpreted as a step toward “normalization” of abnormal perfusion in the frontal cortex in TS, since cerebral blood flow was decreased in this region in TS preOP compared with control subjects. It can be speculated that the increase in cerebral perfusion in frontal regions reflects an improved motor control resulting in a clinical improvement, specifically a tic reduction. Interestingly, this finding is in accordance with a study reporting on an increased perfusion of orbital and anterior medial regions of the frontal lobe bilaterally during successful neuroleptic treatment of tics in young patients with TS (35).

In addition, we found significant decreases of cerebral perfusion during GPi and CM/Voi stimulation compared with SHAM condition in the cerebellum and basal ganglia. As discussed earlier, abnormalities in the cerebellum have even been proposed as being the primary cause of TS (28). It can be hypothesized that both CM/Voi and GPi DBS have an impact on the possibly detrimental excitatory signaling between the cerebellum and the striatum and, therefore, result in clinical improvement.

In contrast to studies investigating less severely affected Tourette patients, we did not observe significant hypoperfusion in the basal ganglia (26, 36, 37). One possible explanation, which has not been investigated thus far, would be a difference in perfusion during the presence of tics vs. that in patients trying to suppress tic activity. Moreover, effects of medication, namely antipsychotic treatment, performed in all but one patient, cannot be ruled out. However, at least in patients with schizophrenia, conflicting results of either decreased or increased brain perfusion in the frontal cortex due to treatment with antipsychotics have been reported (38, 39).

Although our study provides objective data on the impact of DBS electrode implantation and chronic subthreshold stimulation, several limitations have to be addressed. The sample size was rather small. The patient group was heterogeneous with respect to comorbidities, which might hamper the validity of the comparison with the control group. DBS did not result in a statistically significant tic improvement compared with SHAM stimulation. Furthermore, from our data, no conclusion can be drawn whether certain patterns or changes in cerebral perfusion in specific brain areas can be used to predict individual treatment responses to DBS.

## Conclusion

Pathologically reduced frontal cortex perfusion in patients with severe TS can be reversed by GPi and CM/Voi DBS. In addition, both types of DBS reduce abnormally increased cerebellar perfusion. Our data, therefore, provide substantial evidence that, in TS, both GPi and CM/Voi DBS not only result in similar alterations of cerebral blood flow but also cause changes toward normalization.

## Author Contributions

CH: analysis and interpretation of data, drafting of the manuscript, critical revision of the manuscript and for important intellectual content, and statistical analysis. KM-V: study concept and design, acquisition of data, analysis and interpretation of data, drafting of the manuscript, critical revision of the manuscript and for important intellectual content, obtained funding, administrative, technical, and material support, and study supervision. FW: analysis and interpretation of data, statistical analysis. CS: acquisition of data, administrative, technical, and material support. HC: acquisition of data, administrative, technical, and material support. LG: analysis and interpretation of data. FB: analysis and interpretation of data, drafting of the manuscript. JK: study concept and design, analysis and interpretation of data, drafting of the manuscript, critical revision of the manuscript and for important intellectual content, obtained funding, study supervision. GB: study concept and design, acquisition of data, analysis and interpretation of data, drafting of the manuscript, critical revision of the manuscript and for important intellectual content, statistical analysis, administrative, technical, and material support, study supervision.

## Conflict of Interest Statement

The authors declare that the research was conducted in the absence of any commercial or financial relationships that could be construed as a potential conflict of interest.
